# Perioperative and postoperative management of tympanostomy tube insertion: a survey of otorhinolaryngologists in Israel

**DOI:** 10.1007/s00405-024-08964-8

**Published:** 2024-09-06

**Authors:** Dean Dudkiewicz, Efrat Miryam Bismuth, Nir Tsur, Dror Gilony, Roy Hod

**Affiliations:** 1 Department of Otorhinolaryngology-Head and Neck Surgery, Rabin Medical Center, Beilinson Campus, Petah- Tikva, 49100 Israel; 2 Department of Otolaryngology-Head and Neck Surgery, Schneider Children’s Medical Center of Israel, Petach Tikva, Israel; 3 Sackler Faculty of Medicine, Tel Aviv University, Tel Aviv, Israel

**Keywords:** Tympanostomy tubes, Perioperative management, Pediatric otolaryngology, Middle ear infections, Chronic otitis media, Hearing loss, Prophylaxis

## Abstract

**Background:**

Tympanostomy tube insertion is a standard surgical procedure in children to address middle ear infections and effusion-related hearing and speech development issues. Perioperative treatments like ear drops containing antibiotics, steroids, and tube irrigation with saline aim to prevent complications, yet no universal gold standard treatment exists. Despite guidelines, practice preferences among ENT specialists vary, motivating this study to investigate perioperative management practices in Israel.

**Method:**

A survey was distributed among ENT surgeons, collecting data on their main workplace, sub-specialty, preoperative hearing test requirements, tube irrigation practices, tube selection criteria, and timing of tube removal. Distribution and association with main workplaces were examined.

**Results:**

The survey achieved a response rate of 27.33%. Most participants routinely required preoperative hearing tests, with a preference for conducting them within three months prior to surgery (62.2%). Tube irrigation during the procedure was less common among surgeons in the public system (*p* = 0.007). In response to the COVID-19 pandemic, the majority of respondents maintained their established practices (96.3%), while a small proportion (3.7%) adapted by replacing two in-person meetings with one virtual session. Variations in tube removal timing based on the main workplace were noted, with private practitioners opting for earlier removal (*p* = 0.002) and were less permissive in water deprivation practices (*p* = 0.053).

**Conclusion:**

This study provides insights into the practices and preferences of ENT surgeons in tympanostomy tube insertion procedures in Israel. Adherence to standardized practices was observed, with variations influenced by the primary workplace. Despite the COVID-19 pandemic, minimal changes were made to established practices. Further research and consensus are necessary to optimize patient outcomes and develop tailored guidelines in this field.

## Introduction

Myringotomy with insertion of tympanostomy tubes is a common procedure [1, 2] for addressing persistent middle ear infections and chronic otitis media with effusion in children [3, 4] ]. The technique of myringotomy dates back to the 17th century [5], and ventilation tubes were later developed to prevent spontaneous healing and closure of the tympanic membrane [5–7]. Currently, the procedure involves making a small incision in the tympanic membrane, suctioning fluid from the middle ear, and inserting a narrow tube to prevent the incision from shutting down [8]. To prevent local complications [9, 10], various prophylactic treatments are used, such as antibiotic drops, steroid drops, and normal saline drops [1, 11]. Hearing tests are often employed prior to and after the procedure [8, 12, 13]. However, there is no universally accepted gold standard treatment. Previous research has shown mixed results regarding the efficacy of different prophylactic approaches [1, 11, 14]. Despite published guidelines [8], many ENT specialists do not adhere to them [15], indicating a lack of consensus in perioperative management practices.

This study aimed to investigate the preferred perioperative management practices of tympanostomy tube insertion among ENT specialists in Israel, shedding light on potential variations and providing valuable insights for optimizing patient outcomes.

## Methods

### Settings

This qualitative study was conducted in Israel between August and December 2022 to investigate the perioperative treatment management practices of ENT doctors regarding tympanostomy tube insertion procedures. A total of 300 questionnaires were distributed to ENT doctors in Israel via E-mail and WhatsApp. The questionnaires were administered online and comprised a comprehensive survey assessing preoperative investigation protocols and treatment managements before, during, and after the procedure.

Statistical analysis was performed to examine potential associations and differences in treatment management practices based on various factors, including the physician’s gender, experience, main workplace, subspecialty type, previous fellowship and the influence of the COVID-19 pandemic on common practices.

Ethical approval was obtained from the relevant institutional review board, and informed consent was obtained from all participants.

### Statistical analysis

All statistical analyses were carried out using R version 4.1.2 (FSF, Boston, Massachusetts, USA) and R Studio version 1.4 (Posit, Boston, Massachusetts, USA) software. Continuous variables are presented as means and standard deviations. Categorical data were analyzed using Chi-square or Fisher’s exact test, as appropriate. Statistical significance was inferred at *p* < 0.05.

## Results

### Demographics and perioperative management

**Fig. 1:**
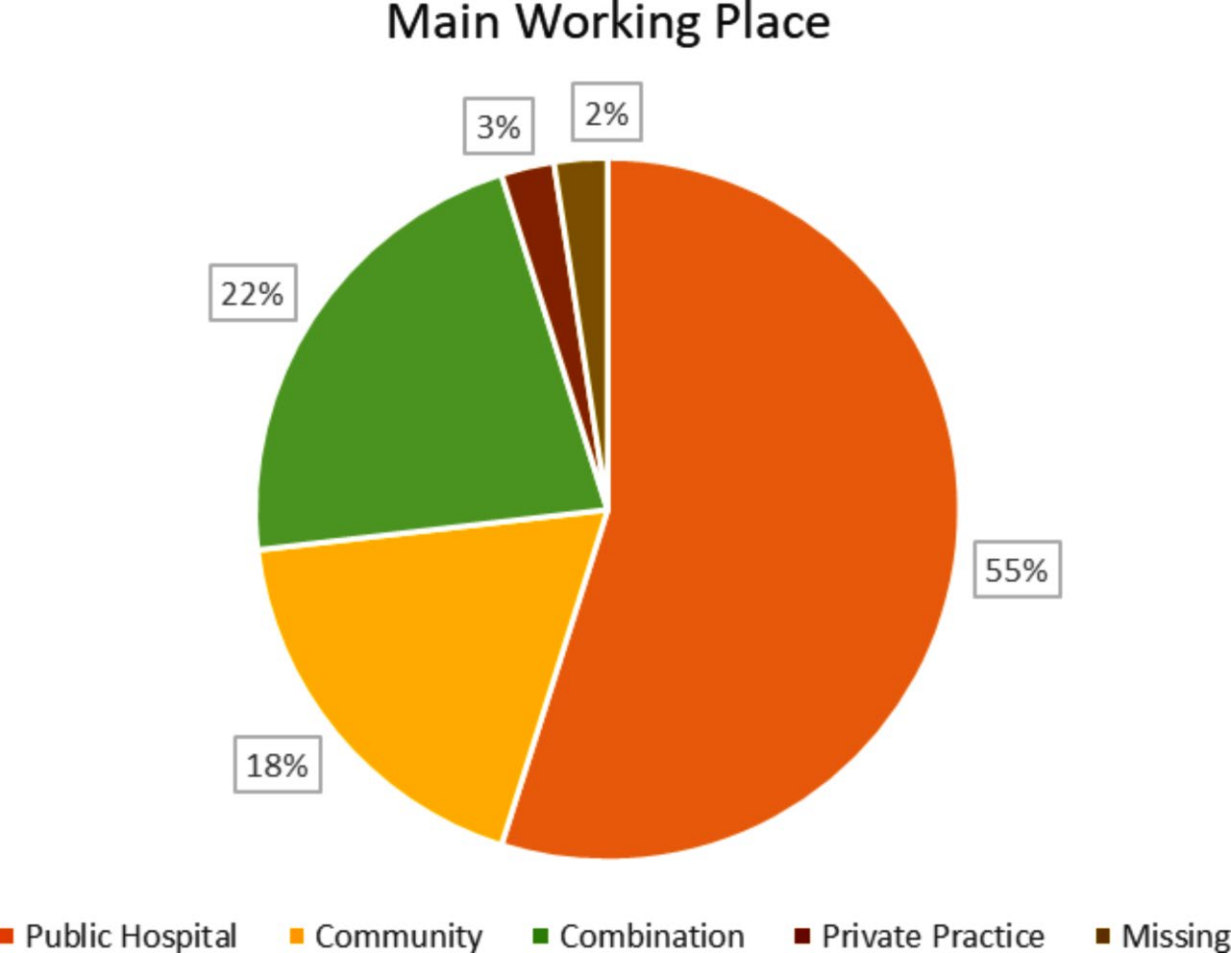
Occupational distribution

In this study, we obtained responses from 82 ENT doctors, representing a response rate of 27.33% out of the 300 questionnaires distributed. The majority of respondents were male. The distribution of gender, residency, and subspecialty status and location (in Israel or abroad) is listed in Table 1. Additionally, their main workplace distribution is outlined in Fig. 1. The rate of missing answers in the survey was up to 11%.

**Table 1:** Ddemographics of 82 pediatric otolaryngologists

	Total (*N* = 82)
Sex (Male)	63 (76.8%)
Professional Status (Attending)	66 (80.5%)
Time From Residency End	
Up to 5 Years	5 (6.1%)
5–10 Years	11 (13.4%)
Over 10 Years	50 (61.0%)
Missing	16 (19.5%)
Sub-Specialty (Yes)	50 (61.0%)
Sub-Specialty Abroad (Yes)	38 (46.3%)
Sub-specialty Field	
Pediatric ENT	21 (25.6%)
Otology	9 (11.0%)
Head & Neck	8 (9.8%)
Nose & Sinuses	7 (8.5%)
Laryngology & Airway	4 (4.9%)
Sleep	1 (1.2%)
Research	1 (1.2%)
Missing/Irrelevant	31 (37.8%)
Surgeon (Yes)	69 (84.1%)

**Fig. 2:**
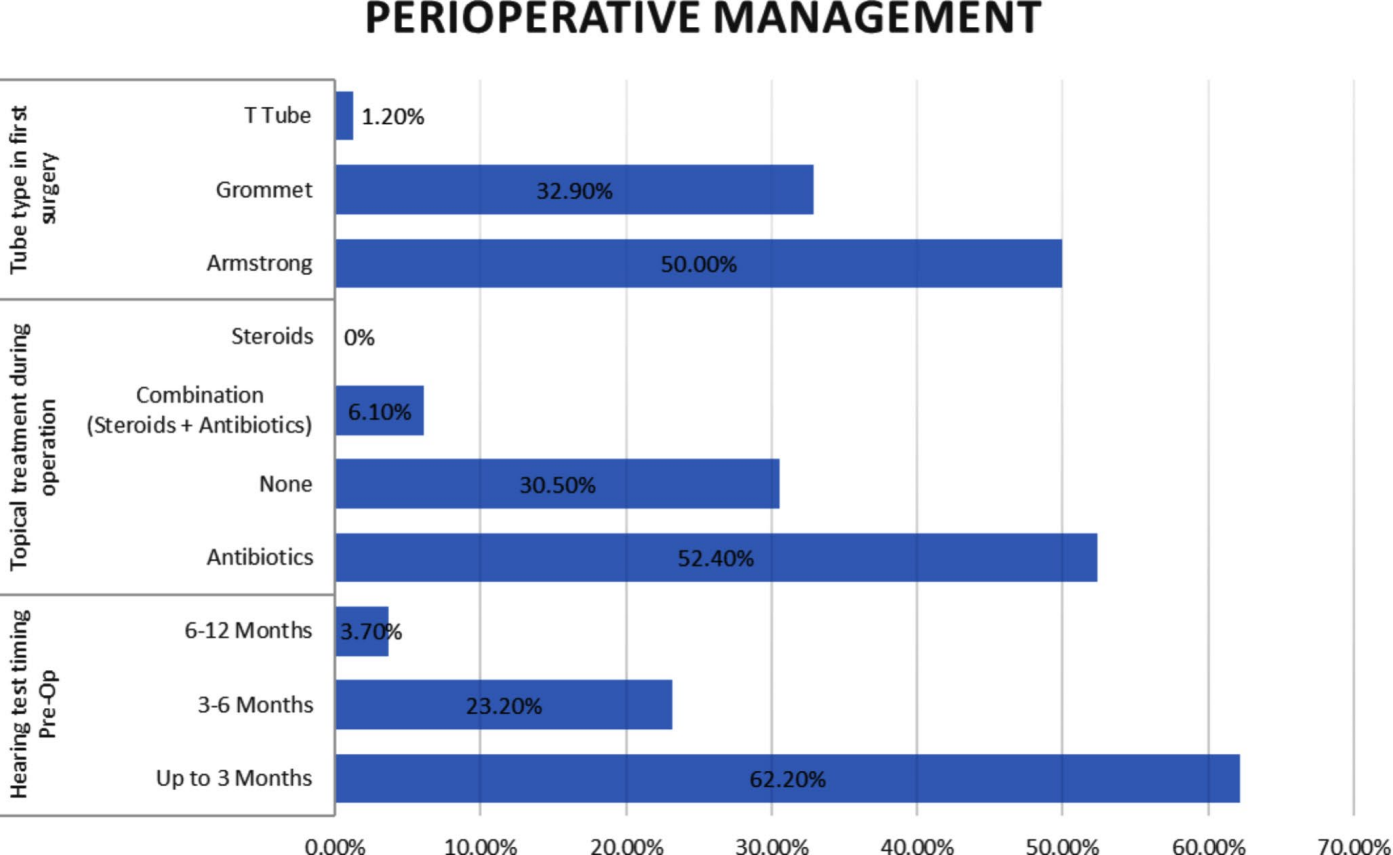
Perioperative management

Regarding perioperative management, a significant proportion of participants (85.4%) reported routinely requiring a preoperative hearing test (62.2% within up to 3 months, 23.2% within 3–6 months, and 3.7% within 6–12 months). Tube irrigation during tympanostomy tube insertion was not performed by the majority of surgeons (68.3%), while 52.4% reported routine use of antibiotic drops during the procedure. Notably, only 19.5% of participants utilized specific tube types for sub-populations. Additional perioperative management practices and preferences can be found in Table 2; Fig. 2.

**Table 2:** Peri-operational management of 82 pediatric otolaryngologists

	Total (*N* = 82)
Routine Hearing Test Pre-Op (Yes)	70 (85.4%)
Tube Irrigation During Operation (Yes)	17 (20.7%)
Specific Tube Use for Sub-Populations (Yes)	16 (19.5%)
Hearing Test During Follow-Up (Yes)	59 (72.0%)
Hearing Test In The Long Term (Yes)	26 (31.7%)
Different Approach Due To COVID-19 (No)	79 (96.3%)
Change In Follow-Up Frequency Due To COVID-19 (No)	72 (87.8%)

**Fig. 3:**
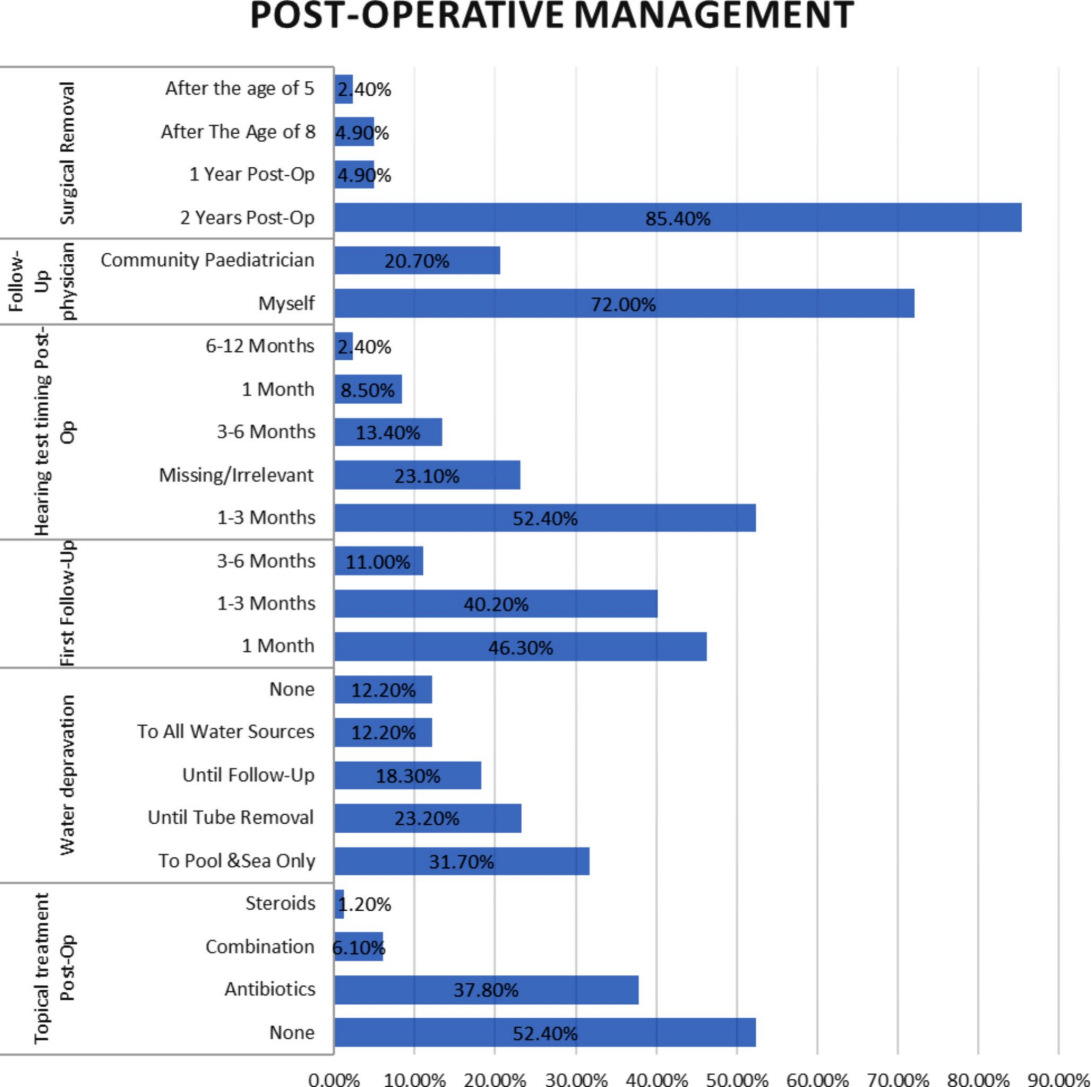
Post-operative management

In terms of post-operative management, a considerable number of participants (52.4%) did not follow a specific protocol, while 37.8% reported routinely administering antibiotic drops. The study unveiled variations in water deprivation practices—18.3% until first follow-up visit, 31.7% limited to pool and sea activities, 12.2% to all water sources, and 23.2% until tube removal. Additionally, it highlighted the necessity for follow-up (46.3% within 1 month, 40.2% 1–3 months, 11% 3–6 months), audiometry timing (with the majority, 52.4%, within 1–3 months), and tube removal protocols (85.4% within 2 years post-op), as illustrated in Fig. 3. Furthermore, the management of otorrhea was preferably done by the surgeon (45%), followed by a community ENT (39%) or community pediatrician (9.8%).

### COVID-19

**Fig. 4:**
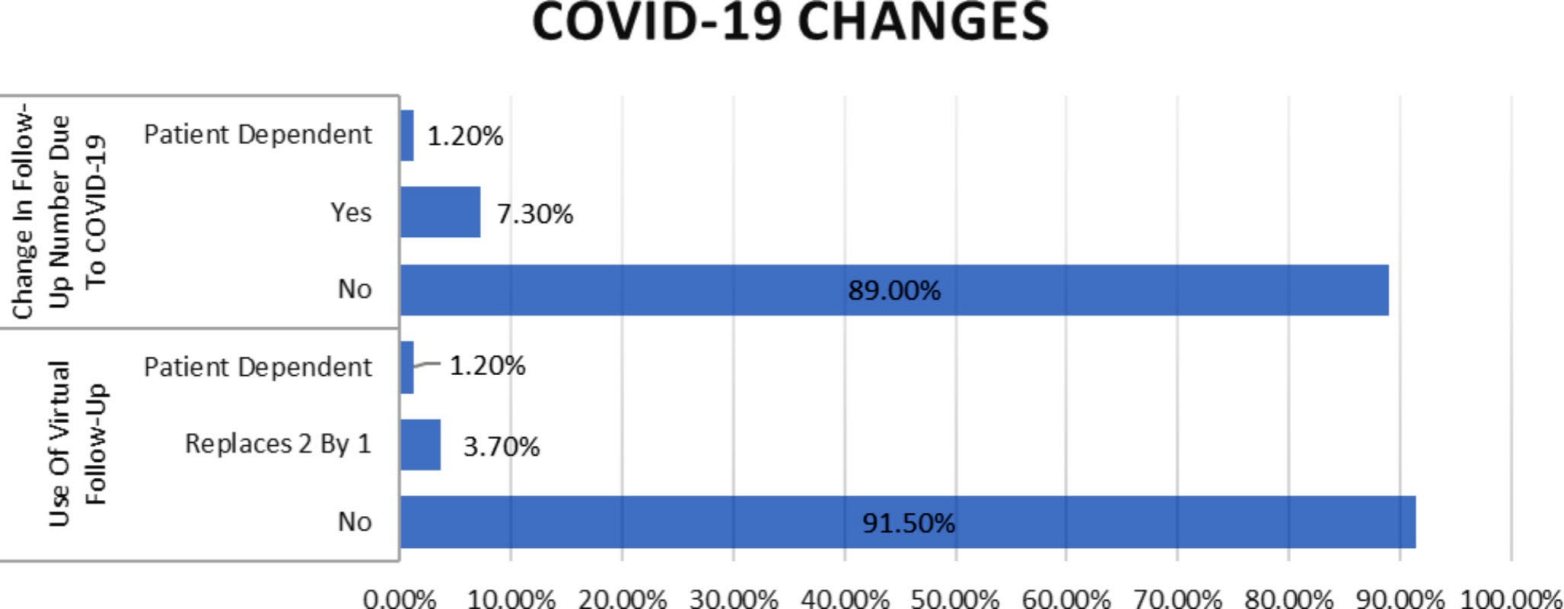
Changes Due-to Covid-19

The majority of respondents, 96.3%, indicated that they did not change their approach to tympanostomy tube management due to the COVID-19 pandemic (Table 2). In terms of virtual follow-up, 91.5% of participants reported not utilizing virtual appointments, while 3.7% replaced two in-person meetings with one virtual meeting, and 1.2% stated that the decision was patient-dependent (Fig. 4).

### Differentiation by main workplace

The results of our study showed significant associations between the main workplace and certain practices in tympanostomy tube insertion procedures. First, a noteworthy association was observed between the main workplace and the use of tube irrigation during ear tube operations (*p* = 0.005). Specifically, ENT doctors in the public system as their main workplace were less likely to utilize tube irrigation during the procedure (Table 3).

**Table 3:** Results by main work place

	Public (*N* = 45)	Community & Private (*N* = 17)	Combination (*N* = 18)	Total (*N* = 82)	*P* Value
Sub-Specialty (Yes)	29 (64.4%)	9 (52.9%)	12 (66.7%)	50 (61.0%)	0.06^†^
Tube Irrigation During Operation (Yes)	7 (15.6%)	7 (63.6%)	3 (16.7%)	17 (20.7%)	0.005^†^
Water Depravation					0.02^†^
Until Follow-Up	11 (24.4%)	2 (11.8%)	2 (11.1%)	15 (18.3%)	
To Pool &Sea Only	17 (37.8%)	2 (11.8%)	7 (38.9%)	26 (31.7%)	
To All Water Sources	4 (8.9%)	4 (23.5%)	2 (11.1%)	10 (12.2%)	
Until Tube Removal	6 (13.3%)	7 (41.2%)	4 (22.2%)	19 (23.2%)	
No	7 (15.6%)	0 (0%)	3 (16.7%)	10 (12.2%)	
Surgical Removal					0.06^†^
1 Year Post-Op	2 (4.4%)	2 (11.8%)	0 (0%)	4 (4.9%)	
2 Years Post-Op	41 (91.1%)	13 (76.5%)	16 (88.9%)	70 (85.4%)	
After the age of 5	0 (0%)	0 (0%)	2 (11.1%)	2 (2.4%)	
After The Age of 8	2 (4.4%)	2 (11.8%)	0 (0%)	4 (4.9%)	
Different Approach Due To COVID-19 (Yes)	0 (0%)	0 (0%)	0 (0%)	0 (0%)	NA
Use Of Virtual Follow-Up					1^†^
Replaces 1 By 1	0 (0%)	0 (0%)	0 (0%)	0 (0%)	
Replaces 2 By 1	2 (4.4%)	1 (5.9%)	0 (0%)	3 (3.7%)	
No	42 (93.3%)	16 (94.1%)	17 (94.4%)	75 (91.5%)	
Patient Dependent	1 (2.2%)	0 (0%)	0 (0%)	1 (1.2%)	
Change In Follow-Up Number Due To COVID-19					0.584^†^
Yes	4 (8.9%)	1 (5.9%)	1 (5.6%)	6 (7.3%)	
No	41 (91.1%)	16 (94.1%)	16 (88.9%)	73 (89.0%)	
Patient Dependent	0 (0%)	0 (0%)	1 (5.6%)	1 (1.2%)	
Change In Follow-Up Frequency Due To COVID-19 (Yes)	4 (8.9%)	0 (0%)	4 (22.2%)	8 (9.8%)	0.09^†^

ENT – Ear, Nose & Throat, OR – Operating Room, COVID 19 – Corona Virus Disease 2019

p-values were calculated using Fisher’s exact test, as well as:

^†^ Chi-square test

p-values < 0.05 were considered significant

Secondly, a noteworthy trend was found between the main workplace and the timing of surgical removal of tubes if they do not fall off naturally (*p* = 0.06). Our findings indicate that ENT doctors in the private and community sectors tend to opt for surgical removal of tubes after one year if they do not fall off. On the other hand, ENT doctors in the public system or combined practices are more likely to remove tubes surgically after two years if they do not fall off.

Furthermore, examination of the association between the primary workplace and sub-specialty involvement revealed interesting trends, though they did not achieve statistical significance (*p* = 0.06). In terms of proportions, physicians within the public system were found to be more inclined to pursue a sub-specialty, whereas practitioners affiliated with private and community settings were less likely to report having a sub-specialty.

In the context of COVID-19 practices, the results were nearly universally unanimous regarding changes in response to the pandemic (100% reported no alterations in practices), the utilization of virtual follow-up (*p* = 0.87), and adjustments in the number of follow-up visits (*p* = 0.58). Conversely, concerning alterations in follow-up frequency due to COVID-19, a discernible trend was observed. While practitioners in private and community practices demonstrated minimal changes in follow-up frequency, those in public and combined practices were more inclined to do so (*p* = 0.09). The detailed distribution and relation to the main workplace can be found in Table 3.

## Discussion

Myringotomy with the insertion of tympanostomy tubes stands as a prevalent intervention for addressing persistent middle ear infections and chronic otitis media with effusion in children [3, 4]. In this study, our aim was to illuminate the contemporary practices and preferences of ENT surgeons in Israel concerning perioperative management for tympanostomy with tube insertion. Our findings present a nuanced picture, revealing both a commitment to standardized practices, particularly evident in the consistent incorporation of audiometric tests, and variations in individual approaches, notably concerning tube irrigation and antibiotic usage. Furthermore, distinctions based on the primary workspace were observed, encompassing differences in tube removal timing, tube irrigation practices, and the pursuit of subspecialties. Interestingly, the impact of the COVID-19 pandemic appeared minimal in our study cohort, reflecting little to no discernible effect on perioperative management practices according to respondent feedback. In our study, several aspects examined have shown consensus in following evidence-based guidelines. Such can be viewed in the case of preoperative hearing tests conducted, as our results have shown that ENT doctors have followed existing guidelines both in terms of the percentage of tests conducted and the recommended timing of the tests [12]. Although factors such as geographical location [16] or main workplace [17] are known to influence the variation in audiometric tests performed, our study did not observe a difference based on the main workplace, possibly due to the fact that the vast majority of patients in our sample were referred to hearing tests. These results are further supported by several studies that have previously showed adherence of ENT physicians to standardized protocols [18, 19], meaning that several key features from those accepted guidelines do in fact get followed. As audiometric tests serves both as an indication for surgery and as a measurement for treatment success, it is reasonable to assume that therefore they are more easily to perform.

While tympanostomy is considered one of the most common surgical procedures done by ENT surgeons [1, 2], our study revealed diverse approaches within both the entire group and specific sub-groups based on workplace. When viewing all the survey respondents, variations were noted in the selection of tube irrigation and the use of antibiotic drops. The results of our study for both were about halved, aligned with the inconclusiveness of evidence regarding antibiotics and treatment failure [20] and the superiority of irrigation over antibiotic use [21]. Therefore, our findings reflect this lack of consensus attributed to this void in standardized, globally accepted, evidence-based protocols.

Additionally, variations were noted based on the main workplace in several fields examined as water deprivation practices and timing of surgical removal of tubes. Public and combined practitioners tended to focus on water deprivation for pool and sea activities only, while community and private practitioners favored water deprivation until tube removal. This practice can be somewhat backed by the minimal decrease in post-op complications attributed to water deprivation [22, 23]. This highlights how the main workplace may influence perioperative practices, as physicians that are compensated per procedure were found more likely to prefer complete water deprivation whereas doctors that work in a public health system were more liberal in a possible exposure to infections. An alternative explanation can be suggested by the academic branching for public hospitals in Israel that requires from surgeons to be constantly up to date with current literature and guidelines that are more liberal with water exposure [24, 25].

Regarding the differentiation of surgically removing of tubes, an association was found between the main workplace and the timing of removal if the tubes did not fall off naturally. ENT doctors in private practices were more inclined to opt for surgical removal after one year, whereas all others were more likely to choose removal after two years. As both practices are seemingly equivalent, according to a recent meta-analysis [26], it is possible that private practitioners are more likely to prefer a quick tube removal as they are reimbursed by procedure and further motivated by the possible complications attributed to retained tube such as tube site infection, permanent tympanic membrane perforation, and cholesteatoma formation [27]. Another plausible explanation may be attributed to a selection bias, given that patients seeking care in public health services are more likely to present with comorbidities and more severe ear conditions [28]. Consequently, patient characteristics, rather than surgeon preferences, may play a pivotal role in dictating perioperative management.

The impact of the COVID-19 pandemic on tympanostomy tube management revealed interesting patterns among respondents. Our findings indicate that while the majority of respondents maintained their established practices for tympanostomy tube management during the COVID-19 pandemic, only a small proportion made minimal adjustments in response to the unique challenges posed by the global health crisis, replacing two in-person meetings with one virtual session to reduce physical contact. This contradicts evidence that surgeons worldwide prefer to make post-op meetings virtually [29], recognizing the importance of direct clinical assessment and patient-provider interaction in the management of tympanostomy tubes. The limited utilization of available alternatives for physical meeting as: virtual appointments, remote otoscopies, in-home hearing test and additional solutions during the pandemic, underscores the significance placed on in-person follow-up visits. Further research and evaluation of the long-term impact of the pandemic on ENT doctors practices may provide valuable insights for future healthcare adaptations and improvements in this area. Additionally, technological developments such as remote endoscopes [30], artificial intelligence [31] and telemedicine [32] will allow physicians to replace physical meetings with virtual ones or can be used to empower and provide greater benefit for the current physical meetings.

While our findings did not reach statistical significance (*p* = 0.06), they reveal noteworthy distinctions in the practices and preferences of ENT doctors based on their primary workplace. Concerning sub-specialty, a majority of doctors in public, private, and combined practices expressed a desire to pursue a sub-specialty, whereas the majority in the community setting did not share this inclination. This suggests that factors associated with the main workplace may exert an influence on ENT doctors’ tendencies toward achieving sub-specialty. Interestingly, our observation contrasts with some studies suggesting that residents inclined toward academic careers, like ones in public and community practices, were more likely to pursue sub-specialty training compared to those leaning toward private practice [33, 34]. A plausible explanation for this phenomenon might be found in the inherent financial implications of pursuing a sub-specialty, considering potential workdays lost versus potential future improved payments. Therefore, it is conceivable that community ENT doctors, who may perceive fewer financial benefits from sub-specialization, are less likely to pursue it.

Looking ahead, the integration of technology, such as telemedicine, remote otoscopies, or in-home hearing tests, holds promising potential for enhancing patient care and follow-up monitoring. These innovative approaches could facilitate more convenient and accessible healthcare services for patients undergoing tympanostomy tube insertion. Additionally, the institutionalization of definite clinical guidelines based on evidence-based practices would provide standardized and optimized care across different healthcare settings.

Several limitations should be considered when interpreting the findings of this study. Firstly, the sample size was relatively small, which may limit the generalizability of the results to a broader population of ENT doctors. Secondly, the study relied on self-report data, which introduces the potential for recall bias or response bias. Lastly, the study was cross-sectional in nature, capturing data at a specific point in time. Longitudinal studies that follow ENT doctors over time would provide a more dynamic perspective on how practices and preferences may evolve or change over the course of their careers.

## Conclusion

In conclusion, this study provides valuable insights into the current practices and preferences of ENT surgeons in Israel regarding perioperative management in tympanostomy tube insertion. While there were variations in individual approaches, adherence to standardized practices was observed. The impact of the COVID-19 pandemic on tympanostomy tube management showed minimal to no change in practice, with limited utilization of virtual appointments. The influence of factors such as the main workplace on ENT practices was observed, underscoring the complexity of the ENT practice landscape. Future studies with larger sample sizes and diverse populations can provide a more comprehensive understanding of the influence of these factors on ENT practices.
